# Direct Oral Anticoagulants in Patients Undergoing Urgent Reperfusion for Nonvalvular Atrial Fibrillation-Related Ischemic Stroke: A Brief Report on Literature Evidence

**DOI:** 10.1155/2019/9657073

**Published:** 2019-02-24

**Authors:** Luca Masotti, Elisa Grifoni, Alessandro Dei, Vieri Vannucchi, Federico Moroni, Grazia Panigada, Costanza Nicotra, Stefano Spolveri, Giancarlo Landini

**Affiliations:** ^1^Internal Medicine, Stroke Unit and Center of Thromboembolic Diseases, San Giuseppe Hospital, Empoli (Florence), Italy; ^2^Internal Medicine, Stroke Unit and Center of Thromboembolic Diseases, Santa Maria Nuova Hospital, Florence, Italy; ^3^Internal Medicine and Stroke Unit, SS. Cosma and Damiano Hospital, Pescia (Pistoia), Italy; ^4^Internal Medicine and Stroke Unit, Borgo San Lorenzo Hospital, Florence, Italy

## Abstract

**Introduction:**

The optimal timing for starting anticoagulation in the early phase of nonvalvular atrial fibrillation (NVAF)-related acute ischemic stroke (AIS) remains a challenge, especially in patients undergoing urgent reperfusion by systemic thrombolysis or mechanical thrombectomy. The aim of our study was to review the literature evidence reporting on safety of direct oral anticoagulants (DOACs) starting in the early phase of NVAF-related AIS undergoing systemic thrombolysis and/or mechanical thrombectomy.

**Materials and Methods:**

We reviewed the PubMed databases searching articles reporting on efficacy and safety of DOACs starting time within two weeks from AIS onset in patients undergoing systemic thrombolysis and/or mechanical thrombectomy.

**Results:**

Three studies were selected, overall including one hundred and six patients (62 females, 58.4%). Median National Institute of Health Stroke Scale (NIHSS) score at hospital admission ranged from 9 to 13 points. Median DOACs starting time ranged from 2 to 6 days. Median CHA_2_DS_2_-VASC score ranged from 4 to 6 points. Follow-up was limited to 14 days in one study, 30 days in another, and 90 days in a third one. Overall, stroke recurrence and/or intracranial bleeding occurred in two patients (1.9%) and no patient died at follow-up.

**Conclusion:**

Small sample size real life studies seem to demonstrate that the introduction of DOACs in the early phase of NVAF-related AIS undergoing urgent reperfusion is efficacious and safe. Prospective RCTs are necessary to confirm these findings.

## 1. Introduction

Preventing stroke recurrences avoiding hemorrhagic transformation (HT) represents the cornerstone of secondary prevention in nonvalvular atrial fibrillation (NVAF) related acute ischemic stroke (AIS). Anticoagulation is the first therapeutic choice in this context [1]. However, the optimal timing for starting anticoagulation in the early phase of NVAF-related AIS remains a challenge, especially in patients undergoing urgent reperfusion by systemic thrombolysis or mechanical thrombectomy.

Evidence for the use of direct oral anticoagulants (DOACs) in the early phase of NVAF-related AIS is lacking, because patients suffering from AIS were excluded from phase III randomized clinical trials (RCTs) on DOACs in stroke prevention in atrial fibrillation (SPAF) [2, 3]. However about 14.000 patients enrolled in phase III RCTs on DOACs in SPAF had suffered from a previous transient ischemic attack (TIA)/stroke and a post hoc analysis of phase III RCTs comparing DOACs versus warfarin in SPAF showed a good efficacy/safety profile of DOACs in this kind of patients [4].

As known, urgent reperfusion by systemic thrombolysis and/or mechanical thrombectomy is associated with better outcomes but, at the same time, with a nonnegligible rate of HT [5]. For this reason, no antithrombotic therapy should be administered in the first 24 hours from systemic thrombolysis [1]. After this time, the optimal timing for starting anticoagulation after systemic thrombolysis and/or mechanical thrombectomy is unknown, especially in patients receiving DOACs. Therefore the aim of our study was to review the literature evidence reporting on safety of DOACs starting in the early phase of NVAF-related AIS undergoing systemic thrombolysis and/or mechanical thrombectomy.

## 2. Materials and Methods

Source data were obtained from the PubMed databases searching articles published until 2018 October, 31 and reporting on DOACs starting time in the early phase from stroke onset. We built our search by combining the terms “oral anticoagulants” with the terms “stroke”, “early”, “atrial fibrillation”, and the terms “oral anticoagulants” with the terms “stroke”, “acute”, “atrial fibrillation” by using the Boolean operator “AND”. Our search strategy was refined by reviewing the bibliography of retrieved articles. The searches were restricted to English language articles, adults (≥18 years), words in title/abstract, DOACs starting time within 14 days from stroke onset, studies reporting on efficacy and safety of DOACs in patients undergoing systemic thrombolysis and/or mechanical thrombectomy.

## 3. Results

**Figure 1:**
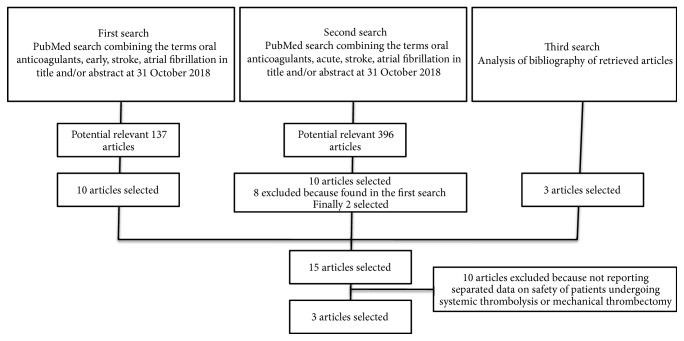
Flow diagram of selected studies.

After searching process (Figure 1), we selected three studies, two retrospective and one prospective observational [6–8]. Overall one hundred and six patients (62 females, 58.4%) were enclosed. Of them ninety-nine patients received systemic thrombolysis (93.3%), three mechanical thrombectomy (2.8%), and four systemic thrombolysis plus mechanical thrombectomy (3.9%). Median or mean age of patients was over 75 years. Median National Institute of Health Stroke Scale (NIHSS) score at hospital admission ranged from 9 to 13 points. Median DOACs starting time was 6 days in two studies and 2 days in another study. Median CHA_2_DS_2_-VASC score ranged from 4 to 6 points. Follow-up was limited to 14 days in one study [8], 30 days in another [7], and 90 days in a third one [6]. Overall, stroke recurrence and/or intracranial bleeding occurred in two patients (1.9%), while no patient died at follow-up.  Table 1 summarized our findings.

## 4. Discussion

The optimal starting time of anticoagulants in the acute phase of NVAF-related AIS remains a clinical dilemma, especially in patients undergoing systemic thrombolysis and/or mechanical thrombectomy. Recently, in the RAF study enrolling 1029 in the early phase of NVAF-related AIS, it was observed that bringing International Normalized Ration (INR) in the range 2.0-3.0 by using vitamin K antagonists (VKAs) between 4 and 14 days was associated with the best 90-day efficacy/safety profile [9]. In this study the mean time for reaching INR ≥ 2 was 12.1 ± 15.8 days [9]. In the RAF study 230 patients (22.3%) underwent urgent reperfusion by systemic thrombolysis and/or mechanical thrombectomy [9]. Of them, 188 patients received anticoagulants after these procedures. 90-day cumulative TIA/stroke or systemic embolism and hemorrhagic events occurred in 27 patients (11.7%). However, data on the starting time of anticoagulants after systemic thrombolysis and/or mechanical thrombectomy were not available [9]. In the VISTA registry enrolling 1644 patients with NVAF-related AIS, 496 patients (30.1%) received anticoagulants or anticoagulants plus antiplatelets after thrombolysis treatment [10]. In the VISTA registry, the incidence of stroke recurrence, symptomatic intracranial bleeding, all-cause mortality, and 90-day modified Rankin Scale (mRS) ≥4 in patients with NVAF-related AIS receiving VKAs was 10.6%, 2.9%, 25.5%, and 46.6%, respectively, while in patients receiving VKAs plus antiplatelets was 6.7%, 1.9%, 17.8%, and 54.5%, respectively [10]. The median starting time of anticoagulants in the VISTA registry was 2 days (IQR 1-4). However, in the VISTA registry, data on starting time and outcome in patients receiving thrombolysis were not available [10].

Evidence for the use of DOACs in the early phase of stroke is lacking because AIS represented a contraindication for the enrollment in phase III RCTs in the context of SPAF. Based on expert opinion, guidelines suggest starting DOACs immediately in patients with NVAF-related TIA, after ≥3, 6-8 and 12-14 days in mild, moderate, or severe NVAF-related AIS, respectively [11]. Despite the absence of strong literature evidence, DOACs seem to represent a great opportunity in patients with NVAF-related AIS, due to their favorable pharmacological and safety profiles. In the latest years a lot of literature evidence about the introduction of DOACs in the early phase of NVAF-related AIS was available. Much recently, Masotti et al. provided a summary of the literature evidence reporting on DOACs in this context [12]. The authors selected fifteen studies, overall enrolling 2920 patients. Median time of starting DOACs ranged from 2 to 8 days, and in twelve of the fifteen selected studies, median or mean starting time was ≤ 7 days [12]. The authors found a 90-day TIA/stroke recurrence, HT/intracranial bleeding, and all-cause mortality incidence of 2.25%, 0.90%, and 1.5% of patients, respectively [12]. Ten of the fifteen selected studies, overall including 2552 patients, reported on percentage of patients undergoing systemic thrombolysis and/or mechanical thrombectomy (32.4%), but only three studies separately reported on efficacy and safety of DOACs in patients receiving systemic thrombolysis or mechanical thrombectomy [12]. In the present overview we focused on these three studies [6–8]. Findings from these three studies should be carefully weighted because of the different size of the studies, the small sample size, the different stroke severity, and follow-up. However, overall the three studies demonstrate that DOACs introduced within one week from urgent reperfusion seem to have a good efficacy/safety profile, at least in the short-term follow-up.

## 5. Conclusion

Literature evidence about efficacy and safety of DOACs after urgent reperfusion by systemic thrombolysis and/or mechanical thrombectomy is poor. Small sample size real life studies seem to demonstrate that the introduction of DOACs in the early phase of NVAF-related AIS undergoing urgent reperfusion is safe. Prospective RCTs are necessary to confirm these findings.

## Figures and Tables

**Table 1:** Summary of selected studies.

Author Reference (year)	Study design	Pts (N)	Females (%)	Age (years, median, IQR or mean ± SD)	Antithrombotic therapy before stroke N (%)	NIHSS at hospital admission (median, IQR)	Large infarct size N (%)	Reperfusion type	DOACs starting (days, median, IQR)	DOAC type	Low dose N (%)	CHA_2_DS_2_-VASC Score after index event (median, IQR)	HAS-BLED Score after index event (median, IQR)	Follow-up	Stroke recurrence and/or intracranial bleeding at follow-up	All cause mortality at follow-up
*Masotti (2018) [6]*	Retrospective	35	23 (65.7%)	84 (78-88)	AP 11 (31.4% VKAs 6 (17.1%) DOACs 0	12 (6-17)	18 (58.2%)^∧^	rtPA 28 Mechanical thrombectomy 3 rtPA plus mechanical thrombectomy 4	6 (4-8)	Dab 9 Riv 3 Api 17 Edo 6	21 (60%)	5 (4-6)	3 (3-3.5)	90-day	1 (2.8%)	0

*Saji (2016) [7]*	Prospective observational	37	21 (56.7%)	76.8 ± 10.2	AP 6 (16.2%) VKAs 10 (27.4%) DOACs 3 (8.1%)	13 (8-19)	14 (37.8%)^∧∧^	rtPA 37	2 (2-3)	Dab 10 Riv 22 Api 5	NA	4 (2-5)	NA	30-day	1 (2.7%	0

*Ritzenthaler* *(2015) [8]*	Retrospective	34	18 (51.4%)	81 (71-85)	NA	9 (6-13)	NA	rtPA 34	6 (4-11)	Dab 9 Riv 25	0 (0%)	6 (4-6)	≥3 26 (76.5%)	14-day	0	0

^∧^ anterior lesions involved the complete territory of the middle cerebral artery (MCA), posterior cerebral artery, or anterior cerebral artery or were in 2 cortical superficial branches of the MCA, in a cortical superficial branch of MCA associated with the MCA deep branch, or in >1 artery territory (e.g., MCA associated with anterior cerebral artery territory); lesions ≥1.5 cm in the brain stem or cerebellum.

^∧∧^infarct size ≥ 3 cm.

N, number; IQR, interquartile range; SD, standard deviation; NIHSS, National Istitute of HealtStroke Scale; DOAC, direct oral anticoagulants; mRS, Modified Rankin Scale; TIA, transient ischemic attack; HT, hemorrhagic transformation; NA, not available; DWI, diffusion-weighted images; AP, antiplatelets; VKAs, vitamin K antagonists; AC, nonspecified anticoagulants; dab, dabigatran; riv, rivaroxaban; api, apixaban; edo, edoxaban; rtPA, recombinant tissue plasminogen activator.
